# The protective effect of hydro-alcoholic extract of mangrove (*Avicennia marina* L.) leaves on kidney injury induced by carbon tetrachloride in male rats

**DOI:** 10.15171/jnp.2016.22

**Published:** 2016-06-05

**Authors:** Naser Mirazi, Seyedeh-Nahid Movassagh, Mahmoud Rafieian-Kopaei

**Affiliations:** ^1^Department of Biology, Faculty of Basic Sciences, Bu-Ali-Sina University, Hamedan, Iran; ^2^Medical Plants Research Center, Shahrekord University of Medical Sciences, Shahrekord, Iran

**Keywords:** Mangrove, *Avicennia marina*, Kidney, Carbon tetrachloride

## Abstract

**Background:**

Materials can cause liver and kidney damage which carbon tetrachloride is one of these substances. Medicinal plants and their essential oils and extracts have been used to a large extent as drugs to better control and management of kidney diseases.

**Objectives:**

The aim of this study was to investigate the effect of hydro-alcoholic extract of *Avicennia marina* leaves in the treatment of renal toxicity induced by carbon tetrachloride.

**Methods:**

Forty-two male rats were randomly divided into 6 groups (n = 7): control (taking normal saline, 0.5 ml/day, intraperitoneally; i.p.), sham (taking olive oil, 0.5 ml/day, i.p., single dose), injury induced by carbon tetrachloride (CCl_4_) 1:1 with olive oil, 0.5 ml single dose, i.p.), treated groups 1, 2 and 3: by carbon tetrachloride 1:1 with olive oil, 0.5 ml single dose and 200 mg/kg, 400 mg/kg or 800 mg/kg *Avicennia marina* extract (AME)/ day for 96 hours, i.p.). By direct blood sampling from the heart, the plasma concentrations of lactate dehydrogenase, blood urea nitrogen (BUN), creatinine and liver enzymes including aspartate aminotransferase (AST), alanine aminotransferase (ALT), and alkaline phosphatase (ALP) were measured. Kidney sections were prepared from all groups and the histological examinations were performed. The results were analyzed using one-way analysis of variance (ANOVA).

**Results:**

The results indicated the significant (*P* < 0.05) increase of serum level of lactate dehydrogenase and liver enzymes of AST, ALT and ALP in the group receiving CCl_4_ compared with the control group, whereas the treatment with hydro-alcoholic extract of mangrove leaves caused a significant (*P* < 0.05) decrease in serum levels of these enzymes in rats treated with carbon tetrachloride compared to the control group. Histological investigation of renal tissue sections showed that the treatment with mangrove leaves extract reduced the necrosis, inflammation and also improved the renal tubules.

**Conclusions:**

Carbon tetrachloride has kidney, liver and cardiac toxicities and mangrove extract is able to inhibit the toxicities of carbon tetrachloride.

**Implication for health policy/practice/research/medical education:**

Mangrove extract is able to inhibit the toxicities induced by carbon tetrachloride and might be used for this purpose.

## 1. Background

Renal toxicity is one of the most common kidney problems and especially occurs when the body is subjected to drugs or chemical reagent (1,2). Among environmental toxins, CCl_4_ dedicated most of conducted studies to itself (3). Due to its geometric symmetry, carbon tetrachloride is a non-polar compound so it can dissolve well in non-polar compounds such as fats, oils and iodine (4). CCl_4_ is a transparent, odorless and non-flammable material. Chronic exposure to carbon tetrachloride cause serious damage to the liver, kidney and increase the risk of cancer (5). Studies showed that CCl_4_ toxicity can result in production of free radicals in most of body tissues such as liver, kidney, lung, testis, heart, brain and blood (6). Carbon tetrachloride poisoning makes the model rats influenced by oxidative stress in many physiological situations. The most important step in the tissue damage caused by CCl_4_ is cytochrome P_450_ transferring system which transfers one electron to the carbon-chlorine bond, generates an anionic radical that as an unstable intermediary eliminates chlorines to produce the central carbon radical and generates trichloromethyl-chloride radical. CCl_3_ radical can be attached to macromolecules or attack to membrane lipids and fatty acids. It can also contrast with oxygen via changing to peroxy tri-chloromethyl (CCl3O2) free radicals which are much more reactive than the former radical and cause similar destruction or damages (6).

Natural antioxidants can protect the body against the adverse effects of carbon tetrachloride and some other toxins (7,8). Medicinal plants have been used to treat various disorders throughout the history of human life, but the use of synthetic drugs was highly prevalent since the middle of last century (9). With the rapid detection of their adverse side effects of synthetic drugs on public health, the trend is increasing for application of medicinal plants as alternatives to synthetic ones (10,11). Mangrove (*Avicennia marina* L.) is belong to Avicenniacea family and genus of Avicennia (12). Mangrove is a lasting plant with perennial green leaves and aerial roots known as pneumatophores which originate from the plant stem and vertically growing out from the mud. This plant is halophytic and is resistant to the salt of sea water and has been scattered in the form of marine tidal forests in some parts of the world (13). This plant has various biological activities and it has been used in traditional medicine for the treatment of skin diseases such as smallpox and ulcers (14). The main active ingredients of *A. marina* are phytoalexins, steroids, carboxylic acids, tannins, flavonoids and terpenes (13). Flavonoid compounds and derivatives are more prevalent in the leaves and twigs of mangrove that can scavenge free radicals (12).

## 2. Objectives

Due to the presence of these compounds and to reduce the side effects of used renal drugs and also to improve the quality of care in the treatment of renal disorders, this study was designed to evaluate the antioxidant effects of *A. marina* leaf extract on CCl_4_ induced toxicity in rat.

## 3. Materials and Methods

### 3.1. Animals

In the intervention study, 42 male Wistar rats weighing 220-250 g were prepared from the Pasteur Institute in Tehran, Iran. All animals were kept in animal’s chamber of Department of Biology, Faculty of Sciences, Bu-Ali Sina University of Hamadan in proper conditions of temperature, humidity and light. Rats had access freely to sufficient food and water. To adapt animals with the new environmental condition, all experiments were carried out after one week.

Mangrove leaves were collected from mangrove forests in the Qeshm Island and its species was accredited by experts of Bu Ali Sina University. The leaves were shade dried and milled. To prepare the hydro-alcoholic extract of mangrove leaves, the powdered leaves were soaked in ethanol (80%) in the refrigerator for 72 hours. The upper fraction was collected and the remained pulps of plant were re-extracted with ethanol (80%) until they became colorless. Then the alcohol was evaporated using rotary evaporator and the obtained extract was concentrated, then placed in the freezer for analysis.

### 3.2. Experimental design

In this study, 42 male rats were randomly divided into 6 groups as follows (n = 7):

Control (taking normal saline, 0.5 mL/day, i.p.), sham (taking olive oil, 0.5 mL/day, i.p. single dose), the group induced toxicity by CCl_4_ (carbon tetrachloride 1:1 with olive oil, 0.5 mL single dose, i.p.), treated groups: (1, 2 and 3 by carbon tetrachloride 1:1 with olive oil, 0.5 mL single dose and 200 mg/kg, 400 mg/kg and 800 mg/kg leaf extract/day for 96 hours, i.p.). All injections were performed intraperitoneally. The blood samples were collected from heart directly and blood urea nitrogen (BUN), serum creatinine, LDH as well as aspartate aminotransferase (AST), alanine aminotransferase (ALT), and alkaline phosphatase (ALP) enzymes were analyzed and the microscopic studies of renal tissues were done.

### 3.3. Ethical issues

The research followed the tenets of the Declaration of Helsinki. The research was approved by ethical committee of Bu-Ali-Sina University. Prior to the experiment, the protocols were confirmed to be in accordance with the Guidelines of Animal Ethics Committee of Bu-Ali-Sina University.

### 3. 4. Statistical analysis

Data analysis were performed using one-way analysis of variance (ANOVA) and the significant level was considered at *P* < 0.05.

## 4. Results

The results obtained in this study indicated that the treatment with hydro-alcoholic extract of mangrove leaves caused a significant (*P* < 0.001) decrease in the serum levels of lactate dehydrogenase and liver enzymes of AST, ALT and ALP in rats treated with carbon tetrachloride. The level of BUN and creatinine in the serum showed a significant decrease in the treated group compared with control group (*P* < 0.001).

**Figure 1:**
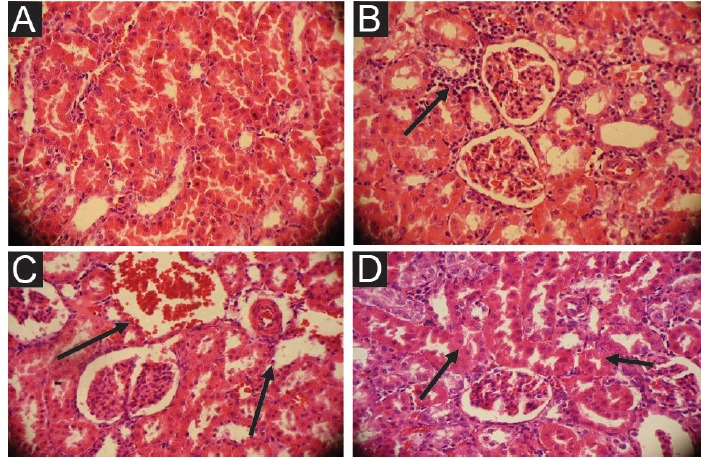
(A) Micrograph of renal tissue in control group. H&E staining . x400. (B) Micrograph of renal tissue in sham group (olive oil 2 mL/kg) . Lymphocyte infiltration (arrow). H&E staining. x400. (C) Micrograph of renal tissue in CCl_4_ – induced group (2 mL/kg) . Injured glomerules & nephron tubules and cell disorders (arrow). H&E staining. x400. (D) Micrograph of renal tissue in CCl_4_ – induced group (2 mL/kg) + mangrove extract treated (800 mg/kg). The repaired renal tissue are seen (arrow). H&E staining. x400.

By exposure to carbon tetrachloride, beside the liver, the kidney tissues showed necrosis and inflammation. Histomorphological study showed that the degree of inflammation in the kidney tissues among the treated groups was different before and after the use of mangrove leaves extract, whereas the control and sham groups had normal kidney tissue (Figures 1A and B). In the control group the leakage of leukocytes around tubules, tubular degradation and destruction the proximal tubules were visible. Also, the blood holes were formed (Figure 1C). In the groups treated with doses of 400 mL/kg and 800 mL/kg, particularly the dose of 800 mL/kg, the inflammatory cells and proximal tubules greatly improved (Figure 1D).

## 5. Discussion

The serum levels of urea and creatinine are often considered as reliable markers for measurement of renal function, thus the increasing of serum levels of these markers are indicators of kidney damage. In this study it was observed that the group treated with CCl_4_ showed a significant increase in serum levels of urea and creatinine following 72 hours of exposure to CCl_4_ (3). A significant reduction in the plasma levels of urea is due to the decreased levels of protein in rats treated with carbon tetrachloride. Reduction of protein levels can be due to the disorder or impaired protein synthesis during stress condition. Creatinine is a product of protein metabolism which is excreted in the urethra by glomerular filtration in higher concentration in comparison with its level in the blood, therefore it can be considered as a sign of kidney dysfunction (15,16).

Histological analysis have shown that some parts of animals’ kidney after treatment with carbon tetrachloride experienced swelling and pooling of blood in the blood vessels of the kidney. Many of the renal tubules damage and lose their apparent functionality and coverage epithelial cells are revealed. The leakage of leukocytes within the tubules could also be observed. A number of glomerular capillaries showed sever sign of the damage such as glomerular accumulation, while other parts had severely damaged (17). The concurrent use of hydroalcoholic extract of mangrove leaves reduced the levels of urea and creatinine in comparison with the group treated with CCl_4_. These results suggest that the hydroalcoholic extract of mangrove leaves poses the strong protective effect on kidney cells against the induced damage by carbon tetrachloride. Probably the mangrove leaf extract due to the antioxidant effects can prohibit the destruction of tubules.

Antioxidant compounds can obstruct the oxidation and peroxidation process of lipids, resulting in reduction of tissue damage and help to continue the blood flow in the kidney and improvement of kidney functionality (18-20). Studies have shown that the antioxidants play an important defensive role against free radicals and besides the scavenging of free radicals, they prevent the increasing of serum levels of AST, ALT, BUN, creatinine, uric acid, cholesterol and blood glucose (21,22). Hence they have protective effects against cardiac, renal liver toxicities (23-25).

## 6. Conclusions

Carbon tetrachloride through the influencing on kidney cells and destruction of tubules significantly increased the serum levels of LDH, BUN, creatinine and liver enzymes of AST, ALP and ALT. The use of mangrove extract significantly decreased the amounts of these enzymes. Due to these changes it can be concluded that the mangrove leaves extract moderated the toxic effect of carbon tetrachloride on the activity of kidney cells. The consumption of hydroalcoholic extract of mangrove leaves at doses of 200, 400 and 800 mL/kg and especially at 400 and 800 mL/kg doses resulted in relative improvement of the adverse effects of CCl_4_. These relative improvements or recovery are likely due to the presence of antioxidant compounds such as flavonoids in mangrove leaves extract.

## Acknowledgments

The authors would like to thank the physiology laboratory experts who helped us with preparation of mangrove leaves extract.

## Authors’ contribution

All Contributed in the design and confirmed the final edition. SNM conducted the research. NM supervised and MRK edited the final version.

## Conflicts of interest

The authors declared no competing interests.

## Funding/Support

This paper was derived from MSc thesis of animal physiology with registered code number: 2125454, 24/sept/2014 which supported by department of biology, faculty of basic sciences, Bu-Ali Sina University, Hamedan, Iran.
